# Association between schizoid tendencies and aggressive behaviors: mediating and moderating influences in childhood trauma and life events among Chinese adolescents

**DOI:** 10.1186/s12991-021-00371-1

**Published:** 2021-12-03

**Authors:** Tingyu Yang, Yuqiong He, Shuxian Wu, Xilong Cui, Xuerong Luo, Jianbo Liu

**Affiliations:** 1 National Clinical Research Center for Mental Disorders, and Department of Psychiatry, Second Xiangya Hospital of Central South University, Changsha, 410011 Hunan China; 2 Department of Child Psychiatry of Shenzhen Kangning Hospital, Shenzhen Mental Health Center, School of Mental Health, Shenzhen University, Shenzhen, 518003 China

**Keywords:** Adolescent, Life events, Aggressive behaviors, Childhood trauma, Schizoid tendencies

## Abstract

**Background:**

This study investigated an association between schizoid tendencies and aggressive behaviors in Chinese adolescents, and explored the underlying mechanism.

**Methods:**

The data of 3094 adolescents aged 12 to 16 years were collected from an epidemiological survey in China. All the subjects or their parents completed the Achenbach’s Child Behavior Checklist, the Childhood Trauma Questionnaire-Short Form, and the Adolescent Self-rating Life Event Checklist (ASLEC).

**Results:**

Relative to the non-schizoid group, adolescents with schizoid tendencies (male or female) showed significantly higher scores for aggressive behaviors, emotional abuse, and ASLEC. Regarding females only, those with schizoid tendencies had significantly higher (lower) scores for physical abuse and emotional neglect (physical neglect). The aggressive behaviors score was predicted by scores for schizoid tendencies (β_male_ = 0.620, β_female_ = 0.638, both *P* < 0.001) and ASLEC (β_male_ = 0.125, β_female_ = 0.061, both *P* < 0.01), and by childhood trauma score (males: emotional neglect [β = 0.045, *P* = 0.021]; females: emotional abuse and sexual abuse [β = 0.118 and − 0.062, both *P* < 0.01]). The ASLEC and childhood trauma scores mediated the association between scores for schizoid tendencies and aggressive behaviors, specifically, emotional neglect (emotional abuse and sexual abuse) in males (females). In females, the interaction between scores for childhood trauma and ASLEC affected the aggressive behaviors score (*P* = 0.023).

**Conclusions:**

Schizoid tendencies are associated with aggressive behaviors among Chinese adolescents. Recent life events and childhood trauma mediated an association between schizoid tendencies and aggressive behaviors. The interaction between childhood trauma and recent life events affected aggressive behaviors in females. Aggressive behaviors in adolescents may be ameliorated by reducing childhood trauma and life events.

## Background

Schizophrenia is a psychotic disorder characterized by hallucinations, delusions, and cognitive deficits, accompanied by impaired executive functions, memory, and speed of mental processing [1]. The prevalence rate of schizophrenia is high (0.28%), with 13.4 million years of life lived with disability to the burden of disease worldwide in 2016 [2]. Aggressive behaviors in individuals with schizophrenia have been linked to increased rates of violent crime [3]. A meta-analysis reported that 33.3% of patients with schizophrenia displayed aggression [4]. Other study reported a 26.6% rate of behavioral aggression among patients with schizophrenia [5]. Psychotic symptoms among people with schizophrenia are a risk factor of aggressive behavior [5, 6]. According to Johns et al. [7], psychotic symptoms exist not only in people with schizophrenia, but also in the general population, including adolescents. A systematic review and meta-analysis of population-based studies reported that psychotic symptoms occurred among 24.5% of children and adolescents [8]. Considering that adolescents in the general population also have aggressive behaviors [9], exploring the association between their aggressive behaviors and psychotic symptoms becomes important.

Based on these studies, we hypothesized that increased aggressive behaviors in the general adolescent population may partly result from experiences that are like psychotic symptoms, such as hearing voices or seeing things that do not exist. To explore the association between aggressive behaviors and psychotic symptoms in adolescents, the present study utilized schizoid and aggressive behavior factors of the Achenbach’s Child Behavior Checklist (CBCL). We hypothesized that schizoid tendencies may be closely associated with aggressive behaviors in Chinese school adolescents.

While an association between schizophrenia and aggressive behaviors has been shown, the underlying mechanism remains unclear. Understanding the mechanism in general adolescent populations has significance for earlier intervention of aggressive behaviors. Some previous similar studies have made attempts in this field. For example, several studies investigated the mediating factors between schizotypy (a broad construct representing a continuum of subclinical and clinical psychosis manifestations [10]) and aggression, including perceived criticism and irritability, peer victimization, peer problems, and low self-esteem [11–13].

Childhood trauma is defined as childhood adverse events such as emotional and physical neglect, or physical, emotional, and sexual abuse [14]. Childhood trauma had been associated with many mental and physical disorders [15–17], making it a public health problem. Toutountzidis et al. [18] found an association between emotional abuse and both positive and negative psychosis-like traits. Marzillier et al. [19] observed that individuals with schizotypy were vulnerable to frequent trauma-related intrusions. Huang et al. [20] also confirmed that individuals at high risk of psychosis had experienced more childhood trauma.

There are also links between childhood trauma and aggression. For example, individuals with childhood trauma had a higher risk of perpetrating violence, according to a systematic review and meta-analysis of prospective studies by Fitton et al. [21]. In China, juvenile violent offenders were more likely to have suffered physical and emotional trauma during early childhood [22]. A systematic review and meta-analysis showed that individuals with psychosis who reported childhood maltreatment were more likely to perpetrate violence [23]. McGuigan et al. [24] found that physical neglect suffered by male adolescents in childhood strongly predicted violent behavior.

Given the above studies, our second hypothesis is that childhood trauma may mediate the association between schizoid tendencies and aggressive behaviors. This exploratory model may help us better understand the underlying mechanism between schizophrenia and aggressive behaviors, and guide early intervention to reduce aggressive behaviors of adolescents with schizoid tendencies.

Recent life events such as loss of family member or exam failure may also be an important mediator between schizoid tendencies and aggressive behaviors. Prior studies support this hypothesis. For example, Raine et al. [13] observed that school children with schizotypal traits were likely to suffer from peer victimization. There is also an association between adverse life events and aggression [25], and Huang et al. [9] found that stressful life events increased the risk of aggression. Based on the above studies, our third hypothesis is that recent life events may be as important as childhood trauma in mediating the association between schizoid tendencies and aggressive behaviors.

Some studies reported that childhood trauma may influence the cognition of recent negative stimulation. For example, Aas et al. [26] found that a history of trauma in childhood was associated with greater differentiation in brain responses to negative facial expressions, compared with positive facial expressions, irrespective of the presence of severe mental disorder. A recent study by Li et al. [27] also showed that childhood trauma increased the effect of a recent life event on depression among Chinese undergraduate students. These studies suggest that recent life events may trigger different brain responses in individuals who had experienced childhood trauma compared with individuals without such history. Therefore, our fourth hypothesis is that the association between schizoid tendencies and aggressive behaviors may be influenced by an interaction between childhood trauma and recent life events.

There is evidence of gender differences in aggression. For example, men demonstrated more physical aggression than did women [28–31]. In addition, the types of childhood trauma experienced by men and women may differ. Toutountzidis et al. [18] reported that men experienced more physical abuse, while women experienced more sexual abuse, relative to the opposite gender. Prior study also explored associations between childhood trauma and psychosis-like traits, which differed by gender: the association between physical abuse and psychosis-like traits was found only in women [18]. Thus, our fifth hypothesis tested in the present study is that the mechanism of association between schizoid tendencies and aggressive behaviors may differ according to gender.

## Method

### Participants

The Ethics Committee of Second Xiangya Hospital of Central South University approved this study. All subjects participated voluntarily. The subjects and their parents or guardians provided signed informed consent.

An epidemiological survey of child and adolescent mental disorders was conducted in Hunan Province, China in 2014 [32]. Overall, 18,778 Achenbach’s Child Behavior Checklist (CBCL) forms were given to students from 13 schools in 2 cities of Hunan province, China. Finally, 17,071 CBCL forms from students (aged 6–16 years) were collected, because 1707 were excluded (for refusal to participate, with > 10% missing data, or age outside the range of 6–16 years). According to the results of the CBCL, 3465 students were CBCL-positive and 13,606 were CBCL-negative. Based on the number of CBCL-positive students (*n* = 3465), CBCL-negative students were randomly selected and matched according to the 1:1 principal rule. Finally, 3465 CBCL-positive students and 3465 CBCL-negative students completed a further questionnaire survey. The questionnaire included the Childhood Trauma Questionnaire-Short Form (CTQ-SF) and the Adolescent Self-rating Life Event Checklist (ASLEC). Considering the age limitation of the CTQ-SF [33], this study only included students aged 12 to 16 years. Finally, the study population comprised 3094 students (aged 12–16 years) who sufficiently completed the CTQ-SF and ASLEC.

### Materials

#### Achenbach’s Child Behavior Checklist (CBCL)

The CBCL, as compiled by Achenbach and Edelbrock [34], is divided into 3 parts. The third part consists of Behavioral Questions that comprise 113 items. In the present study, each item was evaluated by the parents according to the true situation of the child, and was scored on a 3-level scale as 0, 1, or 2. The 113 items were summarized as 8 factors. In the present study, only 2 factors were used, specifically schizoid and aggressive behaviors. For males, schizoid and aggressive behaviors contain 10 and 22 items, respectively. In females, 9 and 22 items are, respectively, included in schizoid and aggressive behaviors. The scores for the schizoid and aggressive behavior factors are each the sum of the scores of their respective items. The higher the score of a schizoid factor or aggressive behaviors factor, the greater the schizoid tendencies or aggressive behaviors, respectively. Su et al. [35] defined the Hunan norm for CBCL, and after a reliability validity test, they concluded that the 12- to 16-year-old norms for boys and girls were accurate for children of Hunan province, China. The test–retest reliability of the behavioral problem portion is 0.77, and the standard validity is 0.61 to 0.76 [35].

#### Childhood Trauma Questionnaire-Short Form (CTQ-SF)

The CTQ-SF consists of 25 items and 3 validity items that can be categorized as 5 factors: emotional abuse, physical abuse, emotional neglect, physical neglect, and sexual abuse [14]. Each item is scored on 5 levels, from never (1 point) to always (5 points). Each factor is scored between 5 and 25 points, with a total score from 25 to 125 points. The CTQ-SF was also shown to have good validity among adolescents [14]. The Chinese version has been validated to assess childhood trauma among Chinese adolescents aged 12 years and older [36]. The total scale Cronbach’s α was 0.73, with Cronbach’s α for emotional abuse, physical abuse, sexual abuse, emotional neglect, and physical neglect ranging from 0.23 to 0.74. The content validity test subscales and total scale correlation coefficients were > 0.5 [36].

#### Adolescent self-rating life event checklist (ASLEC)

The ASLEC is a self-assessment questionnaire consisting of 27 items concerning adverse life events during the previous 12 months that cause psychological reactions in adolescents. Each item first determines whether the event occurred within the last 12 months. If not, the item is scored nil points. If such events occurred, items are rated on a 5-point scale based on their psychological experience for life events. The Chinese version has good internal consistency (0.92), test–retest reliability (0.73), and standard validity [37].

### Statistical analysis

The statistical analyses were conducted using SPSS 26.0 (IBM) with the Process 3.2 plug-in. Based on a previous study, males with scores > 8 points or females with scores > 4 points for schizoid factors were classified as the schizoid tendencies group [38], and the remaining were identified as the non-schizoid tendencies group. The aggressive behaviors score, ASLEC score, and each dimension score of the CTQ-SF in the schizoid tendencies group and non-schizoid tendencies group were compared via independent-sample t-test. Analyses of correlations among the scores for schizoid tendencies, aggressive behaviors, ASLEC, and each CTQ-SF dimension (emotional abuse, physical abuse, sexual abuse, emotional neglect, and physical neglect) were conducted using Pearson’s correlation analysis. Variables that were significantly (*P* < 0.05) associated with the aggressive behaviors score in Pearson’s correlation analysis were included in the linear regression analysis as independent variables of the regression. The Process 3.2 plug-in was used to perform mediation analysis with bootstrapping to 5000 (Selected model 4) and moderation analysis (Selected model 3). In the mediation model, if the 95% confidence interval did not include zero, it was considered statistically significant. For other tests, statistical significance was set at *P* < 0.05.

## Results

### Sociodemographic characteristics of adolescents

Of the 3094 participants, 1586 (51.26%) were male and 1508 (48.74%) were female (aged 13.57 ± 1.29 and 13.73 ± 1.31 years, respectively). Overall, 1817 (58.73%) had siblings; and 1508 (48.74%), 747 (24.14%), and 676 (21.85%) lived in rural, urban, and urban–rural areas. These young people considered their family’s economic status as average (2465 [79.67%]), relatively wealthy (452 [14.61%]), or financially constrained (177 [5.72%]).

### Scores for aggressive behaviors, ASLEC, and each dimension of the CTQ-SF, of the groups with or without schizoid tendencies

In males, the scores for aggressive behaviors, emotional abuse, and ASLEC were significantly higher in the group with schizoid tendencies compared with the non-schizoid tendencies group (Table 1). In females, the group with schizoid tendencies had significantly higher scores for aggressive behaviors, emotional abuse, physical abuse, emotional neglect, and ASLEC compared with the non-schizoid tendencies group, while the score for physical neglect was significantly lower (*P* = 0.005).

**Table 1:** Scores for aggressive behaviors, 5 dimensions of the CTQ-SF, and ASLEC between the schizoid tendencies and non-schizoid tendencies groups by gender

	Group	Male score	*t*	*P*	Female score	*t*	*P*
Aggressive behaviors	Schizoid tendencies	19.600 ± 6.687	13.693	< 0.001	15.939 ± 7.036	25.062	< 0.001
	Non-schizoid tendencies	7.321 ± 6.818			5.508 ± 5.232		
Emotional abuse	Schizoid tendencies	8.067 ± 3.626	3.137	0.003	7.733 ± 3.248	6.944	< 0.001
	Non-schizoid tendencies	6.585 ± 2.518			6.427 ± 2.007		
Physical abuse	Schizoid tendencies	6.650 ± 3.118	1.366	0.177	5.909 ± 2.196	3.821	< 0.001
	Non-schizoid tendencies	6.094 ± 2.403			5.424 ± 1.339		
Sexual abuse	Schizoid tendencies	6.067 ± 2.887	1.492	0.141	5.367 ± 1.780	1.896	0.059
	Non-schizoid tendencies	5.506 ± 1.887			5.173 ± 0.979		
Emotional neglect	Schizoid tendencies	12.783 ± 5.764	1.863	0.067	11.612 ± 5.348	4.392	< 0.001
	Non-schizoid tendencies	11.375 ± 5.126			10.207 ± 4.296		
Physical neglect	Schizoid tendencies	9.600 ± 3.761	− 0.229	0.819	8.948 ± 3.397	− 2.842	0.005
	Non-schizoid tendencies	9.702 ± 3.366			9.529 ± 3.246		
ASLEC	Schizoid tendencies	26.967 ± 16.601	5.424	< 0.001	21.373 ± 13.890	10.901	< 0.001
	Non-schizoid tendencies	15.909 ± 15.444			12.077 ± 12.956		

The schizoid tendencies and non-schizoid tendencies groups comprised, respectively, 60 and 156 males and 330 and 1178 females

### Correlations between scores for schizoid tendencies and scores for aggressive behaviors, ASLEC, and each dimension of the CTQ-SF

In both the males and females, the score for schizoid tendencies positively correlated with, respectively, the scores for aggressive behaviors, ASLEC, and the 4 dimensions of the CTQ-SF (i.e., emotional abuse, physical abuse, sexual abuse, and emotional neglect; all *P* < 0.001; Table 2). The aggressive behaviors score significantly correlated with the ASLEC score and each of the scores of the 4 dimensions of the CTQ-SF. The ASLEC score positively correlated with the scores of the 4 dimensions of the CTQ-SF, and negatively correlated with the physical neglect score (*P* < 0.001).

**Table 2:** Correlations for the scores of schizoid tendencies, aggressive behaviors, 5 dimensions of the CTQ-SF, and ASLEC in males and females (r)

Gender		Schizoid tendencies	Aggressive behaviors	Emotional abuse	Physical abuse	Sexual abuse	Emotional neglect	Physical neglect	ASLEC
Male	Schizoid tendencies	1							
	Aggressive behaviors	0.656**	1						
	Emotional abuse	0.228**	0.195**	1					
	Physical abuse	0.146**	0.118**	0.640**	1				
	Sexual abuse	0.117**	0.077*	0.467**	0.477**	1			
	Emotional neglect	0.090**	0.113**	0.272**	0.240**	0.169**	1		
	Physical neglect	− 0.004	− 0.045	0.219**	0.200**	0.225**	0.402**	1	
	ASLEC	0.267**	0.293**	0.356**	0.307**	0.157**	0.127**	− 0.088**	1
Female	Schizoid tendencies	1							
	Aggressive behaviors	0.689**	1						
	Emotional abuse	0.240**	0.299**	1					
	Physical abuse	0.148**	0.183**	0.518**	1				
	Sexual abuse	0.084**	0.056*	0.330**	0.438**	1			
	Emotional neglect	0.132**	0.164**	0.346**	0.261**	0.153**	1		
	Physical neglect	− 0.045	− 0.029	0.174**	0.079**	0.157**	0.336**	1	
	ASLEC	0.318**	0.311**	0.363**	0.290**	0.133**	0.151**	− 0.140**	1

*P < 0.05; **P < 0.001

### Linear regression model to predict aggressive behaviors score

Based on the results of the correlation analyses, only statistically significant variables (*P* < 0.05) were included for the linear regression analysis (Table 3). In males, the aggressive behaviors score was significantly and positively predicted by the schizoid tendencies score (β = 0.620, *P* < 0.001), emotional neglect score (β = 0.045, *P* = 0.021), and ASLEC score (β = 0.125, *P* < 0.001).

**Table 3:** Linear regression model to predict aggressive behaviors score in males and females

Gender	Variables	B	SE	β	*t*	*P*
Male	Schizoid tendencies	1.687	0.053	0.620	31.551	< 0.001
	Emotional abuse	0.066	0.073	0.024	0.911	0.362
	Physical abuse	− 0.079	0.075	− 0.027	− 1.053	0.292
	Sexual abuse	− 0.079	0.082	− 0.021	− 0.970	0.332
	Emotional neglect	0.063	0.027	0.045	2.311	0.021
	ASLEC	0.057	0.009	0.125	6.049	< 0.001
Female	Schizoid tendencies	1.520	0.046	0.638	32.781	< 0.001
	Emotional abuse	0.351	0.069	0.118	5.105	< 0.001
	Physical abuse	0.128	0.103	0.028	1.240	0.215
	Sexual abuse	− 0.367	0.121	− 0.062	− 3.028	0.003
	Emotional neglect	0.049	0.030	0.032	1.623	0.105
	ASLEC	0.032	0.011	0.061	2.971	0.003

In females, the positive predictors of the aggressive behaviors score were the scores of the following: schizoid tendencies (β = 0.638, *P* < 0.001), emotional abuse (β = 0.118, *P* < 0.001), and ASLEC (β = 0.061, *P* = 0.003). However, the sexual abuse score (β = – 0.062, *P* = 0.003) was significant negative predictor of the aggressive behaviors score.

### Mediation analysis for the association between schizoid tendencies score and aggressive behaviors score

In males, the ASLEC score and emotional neglect score mediated the association between the scores for schizoid tendencies and aggressive behaviors, as 95% confident intervals (CIs) did not include zero (Table 4). The scores for emotional abuse, physical abuse, and sexual abuse did not mediate the association between the scores for schizoid tendencies and aggressive behaviors, as the 95% CIs did include zero (Table 4). In females, the scores for ASLEC, emotional abuse, and sexual abuse mediated the association between the scores for schizoid tendencies and aggressive behaviors, as the 95% CIs did not include zero. The scores for physical abuse and emotional neglect did not mediate the association between the scores for schizoid tendencies and aggressive behaviors, as the 95% CIs did include zero (Table 4).

**Table 4:** Indirect paths for the scores of ASLEC and each dimension of the CTQ-SF mediating the association between scores for schizoid tendencies and aggressive behaviors in males and females

Gender	Paths	Effect	Boot SE^†^	Boot LLCL^†^	Boot ULCL^†^
Male	Schizoid tendencies → ASLEC → aggressive behaviors	0.0907	0.0194	0.0467	0.1229
	Schizoid tendencies → emotional abuse → aggressive behaviors	0.0148	0.0188	− 0.0175	0.0562
	Schizoid tendencies → physical abuse → aggressive behaviors	− 0.0095	0.0119	− 0.0338	0.0127
	Schizoid tendencies → sexual abuse → aggressive behaviors	− 0.0045	0.0080	− 0.0216	0.0107
	Schizoid tendencies → emotional neglect → aggressive behaviors	0.0163	0.0073	0.0041	0.0322
Female	Schizoid tendencies → ASLEC → aggressive behaviors	0.0429	0.0169	0.0123	0.0777
	Schizoid tendencies → emotional abuse → aggressive behaviors	0.0691	0.0180	0.0362	0.1079
	Schizoid tendencies → physical abuse → aggressive behaviors	0.0096	0.0097	− 0.0068	0.0314
	Schizoid tendencies → sexual abuse → aggressive behaviors	− 0.0120	0.0075	− 0.0306	− 0.0014
	Schizoid tendencies → emotional neglect → aggressive behaviors	0.0119	0.0073	− 0.0010	0.0278

^†^Boot: bootstrapping; LLCL: lower level confidence limit; ULCL: upper level confidence limit

### Interactions between childhood trauma score and ASLEC score moderated the association between the schizoid tendencies score and aggressive behaviors score

This study further tested the effect of interaction between childhood trauma score and ASLEC score to moderate the association between the schizoid tendencies score and aggressive behaviors score (Table 5). The results showed that interactions between the childhood trauma and the ASLEC scores did not affect the association between the scores for schizoid tendencies and aggressive behaviors, either the males or females. However, in females only, there was an interaction effect shown between the scores for childhood trauma and ASLEC that influenced the score for aggressive behaviors (*P* = 0.023).

**Table 5:** Interaction effect between childhood trauma score and ASLEC score moderating the association between scores for schizoid tendencies and aggressive behaviors

Gender	Variables	Coeff	SE	*t*	*P*
Male	Schizoid tendencies	2.090	0.301	6.937	0.000
	ASLEC	0.110	0.047	2.346	0.019
	Schizoid tendencies *ASLEC	− 0.013	0.010	− 1.293	0.196
	Childhood trauma	0.032	0.030	1.063	0.288
	Schizoid tendencies *Childhood trauma	− 0.006	0.007	− 0.825	0.410
	ASLEC* Childhood trauma	− 0.001	0.001	− 0.696	0.487
	Schizoid tendencies * ASLEC* Childhood trauma	0.000	0.000	0.611	0.541
Female	Schizoid tendencies	1.388	0.282	4.918	0.000
	ASLEC	− 0.071	0.057	− 1.252	0.211
	Schizoid tendencies *ASLEC	0.012	0.010	1.266	0.206
	Childhood trauma	0.014	0.034	0.410	0.682
	Schizoid tendencies *Childhood trauma	0.006	0.007	0.792	0.429
	ASLEC* Childhood trauma	0.003	0.002	2.282	0.023
	Schizoid tendencies * ASLEC* Childhood trauma	0.000	0.000	− 1.722	0.085

## Discussion

### Interpretation of differences in scores for ASLEC and each dimension of the CTQ-SF between the groups with and without schizoid tendencies

This study determined that both the female and male adolescents in the schizoid tendencies group had suffered more emotional abuse and recent life events, compared with adolescents without schizoid tendencies (control). In addition, the females in the schizoid tendencies group were more likely to have experienced physical abuse and emotional neglect. These results are consistent with other reports in which individuals with schizoid personalities were more likely to experience adverse life events [39] and childhood trauma [20], compared with healthy control groups. In addition, Kelleher et al. [40] found that patients with psychotic disorders were more likely to have experienced more severe childhood maltreatment than healthy individuals. A meta-analysis showed that childhood maltreatment was quite common among people at high risk of psychosis, and childhood trauma was strongly associated with psychotic status [41]. People with  schizoid tendencies are more likely to live  with family members with mental disorders [42]. It is well known that people with mental disorders are more prone to have aggression and abusive behaviors [43]. In addition, people with schizotypal personality disorder were found to display an abnormal subjective experience of emotion, such as lack of social pleasure [44]. This makes them more difficult to be understood in social settings, and thus more prone to childhood maltreatment [45].

The results of the present study also indicated that childhood trauma differed between genders in the schizoid tendencies group, and occurred more frequently in females. This is consistent with previous researches, which found that childhood maltreatment appeared to be more profound in the female general population [4, 46] and patients with schizophrenia [45]. The present study is the first study to report differences between genders in adolescents with schizoid tendencies. In addition, an interesting result was that females with schizoid tendencies showed a lower score for physical neglect than those without schizoid tendencies. It may be that parents pay more attention to the physical needs of females who displayed schizoid tendencies, because of their unusual characteristics. There is no relevant research currently to support this result, and further research is needed.

### Schizoid tendencies is a predictor of aggressive behaviors

In the present study, adolescents in the schizoid tendencies group achieved higher aggressive behaviors score compared with the control group, and linear regression analysis suggested that schizoid tendencies score could predict the aggressive behaviors score. This is consistent with most other similar studies, in which aggression was common in people with schizotypy [12, 13]. Schizoid tendencies may predict aggressive behaviors because schizoid symptoms directly induce aggressive behaviors, and aggressive behaviors are indirectly induced by interpersonal problems. For example, patients with schizophrenia may display aggressive behaviors under the influence of command auditory hallucination [47, 48]. Individuals with schizoid tendencies are likely to feel insecure under the influence of delusion, which leads to hostility and aggressive behaviors [49]. In addition, people with schizoid personality and schizophrenia may suffer from social cognition deficits [50] and they are more likely to misunderstand the intentions of others while communicating, which can result in aggressive behaviors [51]. Wong et al. [12] found that suspected schizotypy in children and adolescents was associated with reactive aggression, mainly because of problems with peers and low self-esteem. Raine et al. [13] reported that adolescents with schizotypy elicit peer victimization, which also results in aggressive behaviors. Other study report that people with schizophrenia are more likely to display violent behaviors due to substance abuse [52]. Therefore, interventions such as social skills training and peer education may be recommended for adolescents with schizoid tendencies to prevent aggressive behaviors.

### Life events and types of childhood trauma predict aggressive behaviors differently by gender

In the present study, the linear regression analysis revealed that the life events score could positively predict the aggressive behaviors score. Those who experienced recent life events were more likely to display aggressive behaviors. These results are consistent with previous articles [53, 54]. Ettekal et al. [55] found that higher levels of peer rejection increased the likelihood of aggressive behaviors, and higher quality  of friendships could relieve the negative effect of peer rejection.

In the present study, emotional neglect was a positive predictor of aggressive behaviors in males, and emotional abuse was a positive predictor of aggressive behaviors in females. This suggests a difference in gender response to childhood trauma that affects aggressive behaviors. A study conducted in Ontario Canada found that adolescents exhibited aggressive behaviors because of neglect, specifically when their caregivers no longer played a caregiving role [56]. Another study showed that chronic neglect at an early age predicted aggressive behaviors in later life, and compared with females, males were more likely to show aggressive behaviors [57]. McGuigan et al. [24] found that neglect was a powerful predictor of aggressive behaviors among male adolescents, after controlling for domestic violence and physical abuse. The above studies are consistent with our results. In our study, emotional abuse was a positive predictor of aggressive behaviors in female adolescents, not in males. One possible reason is that males suffer less rumination after emotional abuse compared with females [58, 59], while lower rumination can predict an decrease in aggressive behaviors over time [60]. 

Interestingly, sexual abuse was negative predictor of aggressive behaviors in female adolescents, which is inconsistent with another study [61]. The reason for our result  may be because females were more distressed and more prone to self-blame, developing coping strategies include withdrawal and attempted amnesia after sexual abuse than males [62]. More seriously, females were more likely to develop internalized problems such as suicidal ideation and depression, rather than aggressive behavior after suffering sexual abuse [63].

### Mediation model of schizoid tendencies and aggressive behaviors

In accord with previous studies [64, 65], the present research showed a close association between schizoid tendencies, childhood trauma, recent life events, and aggressive behaviors. These results are consistent with our second and third hypotheses, that childhood trauma may mediate the link between schizoid tendencies and aggressive behaviors, and that recent life events may be as important as childhood trauma in influencing the association between schizoid tendencies and aggressive behaviors.

Adolescents with schizoid features are more likely to experience childhood maltreatment and adverse life events [39], which in turn is a documented risk factor of aggression. Among the males of the present study, emotional neglect mediated the association between schizoid tendencies and aggressive behaviors, and in females, emotional and sexual abuse mediated this association. Sexual abuse had a negative mediating effect between schizoid tendencies and aggressive behaviors among the females, probably because sexual abuse may mainly induce internalized problems rather than aggressive behaviors [63]. Secondly, the mediating effect of sexual abuse in this study is rather small, which may be caused by sampling error. This needs to be addressed further in the follow-up study.

Reducing childhood maltreatment and life events in adolescents with schizoid tendencies may be crucial to reduce their aggressive or violent behaviors, whereas parental love may be important protective factors. For example, Family Attachment Narrative Therapy was used to heal the bad effect of childhood maltreatment [66]. If adolescents with schizoid tendencies are raised in an abusive environment, then timely interventions, such as behavior management and therapy, should be implemented at home and at school.

### Moderation model of schizoid tendencies and aggressive behaviors

The present results showed that the association between schizoid tendencies and aggressive behaviors was not influenced by an interaction between childhood trauma and recent life events. There was an interaction effect between childhood trauma and recent life events that influenced aggressive behaviors in the females. This result is in accord with Beckmann’s study [67], which included 8458 adolescents in Germany: those who experienced parent-to-child physical violence in early life were more likely to display aggression when suffering from recent peer bullying. However, the interaction between childhood trauma and recent life events affected aggressive behaviors only in the females, but not in males. This is the first time that this gender difference is reported.

Functional magnetic resonance imaging studies have shown that maltreatment could lead to changes in the amygdala of females [68]. These changes can result in abnormal activation when adolescents experience adverse life events, leading to reactive aggressive behaviors [69]. In addition, another study found that childhood maltreatment can lead to dysfunction of the hypothalamus–pituitary–adrenal axis (HPA) [70], while aggressive behavior is closely related to HPA function [71]. The present findings suggest that females who experience childhood trauma are highly affected by recent life events and develop aggressive behaviors. This may be because girls are more prone to attenuation of the HPA axis after experiencing childhood maltreatment [72].

## Limitations

There are several limitations in this study. First, the CTQ-SF and ASLEC are retrospective questionnaires, which can cause recall bias. Second, this was a cross-sectional study, so it is impossible to infer the causal association between schizoid tendencies and aggressive behaviors. In addition, there were overlaps between childhood maltreatment and recent life events. The ASLEC evaluated the past 12 months, which may coincide with the existence of childhood trauma in an investigation of adolescents. There are also childhood adversities that were not included in this analysis, such as witnessing community violence [73]. Finally, the CTQ-SF method assessed only the severity of 5 different types of childhood trauma, and did not include the time of occurrence, frequency, or duration of maltreatment. Previous studies have shown that the earlier the childhood trauma begins, the longer it lasts, and the greater its effect on mental health [74]. In future studies, these missing potential factors should be addressed. A future prospective follow-up study should focus on the causal association between adolescent life events, childhood trauma, schizoid tendencies, and aggressive behaviors. In addition, intervention studies can be performed to verify the association and reduce the negative effects of childhood trauma and recent life events.

## Conclusion

This is the first study to investigate the association between childhood trauma, recent life events, schizoid tendencies, and aggressive behaviors among Chinese adolescents. It was found that adolescents with schizoid tendencies were more likely to experience childhood trauma, recent life events, and have aggressive behaviors, compared with those without schizoid tendencies. Recent life events, childhood trauma (specifically emotional neglect in males and emotional abuse in females), and schizoid tendencies independently increased aggressive behaviors. Recent life events and childhood trauma mediated the association between schizoid tendencies and aggressive behaviors in male and female adolescents, and the interaction between childhood trauma and recent life events affected the aggressive behaviors of females. Therefore, reducing childhood trauma (especially emotional neglect in males and emotional abuse in females) and adverse life events may ameliorate aggressive behaviors among adolescents with schizoid tendencies.

## Data Availability

The raw data required to reproduce these findings cannot be shared at this time as the data also form part of an ongoing study.

## Abbreviations

ASLEC: Adolescent Self-rating Life Event Checklist
CBCL: Achenbach Child Behavior Checklist
CTQ-SF: Childhood Trauma Questionnaire-Short Form
HPA: Hypothalamus–pituitary–adrenal axis
